# Beat the stress: breeding for climate resilience in maize for the tropical rainfed environments

**DOI:** 10.1007/s00122-021-03773-7

**Published:** 2021-02-16

**Authors:** Boddupalli M. Prasanna, Jill E. Cairns, P. H. Zaidi, Yoseph Beyene, Dan Makumbi, Manje Gowda, Cosmos Magorokosho, Mainassara Zaman-Allah, Mike Olsen, Aparna Das, Mosisa Worku, James Gethi, B. S. Vivek, Sudha K. Nair, Zerka Rashid, M. T. Vinayan, AbduRahman Beshir Issa, Felix San Vicente, Thanda Dhliwayo, Xuecai Zhang

**Affiliations:** 1 International Maize and Wheat Improvement Center (CIMMYT), ICRAF Campus, UN Avenue, Gigiri, P.O.Box 1041–00621, Nairobi, Kenya; 2 CIMMYT, P.O. Box MP163, Harare, Zimbabwe; 3 CIMMYT, ICRISAT Campus, Patancheru, Greater Hyderabad, Telangana India; 4 CIMMYT, Kathmandu, Nepal; 5 CIMMYT, El Batan, Texcoco, Mexico, DF Mexico

## Abstract

**Key message:**

Intensive public sector breeding efforts and public-private partnerships have led to the increase in genetic gains, and deployment of elite climate-resilient maize cultivars for the stress-prone environments in the tropics.

**Abstract:**

Maize (*Zea mays* L.) plays a critical role in ensuring food and nutritional security, and livelihoods of millions of resource-constrained smallholders. However, maize yields in the tropical rainfed environments are now increasingly vulnerable to various climate-induced stresses, especially drought, heat, waterlogging, salinity, cold, diseases, and insect pests, which often come in combinations to severely impact maize crops. The International Maize and Wheat Improvement Center (CIMMYT), in partnership with several public and private sector institutions, has been intensively engaged over the last four decades in breeding elite tropical maize germplasm with tolerance to key abiotic and biotic stresses, using an extensive managed stress screening network and on-farm testing system. This has led to the successful development and deployment of an array of elite stress-tolerant maize cultivars across sub-Saharan Africa, Asia, and Latin America. Further increasing genetic gains in the tropical maize breeding programs demands judicious integration of doubled haploidy, high-throughput and precise phenotyping, genomics-assisted breeding, breeding data management, and more effective decision support tools. Multi-institutional efforts, especially public–private alliances, are key to ensure that the improved maize varieties effectively reach the climate-vulnerable farming communities in the tropics, including accelerated replacement of old/obsolete varieties.

## Introduction

Maize is one of the most important and widely grown crops in the world. While maize is a major source of feed and industrial products in the high-income countries, it provides food, feed, and nutritional security in the world’s poorest regions in sub-Saharan Africa (SSA), Asia, and Latin America. The crop accounts for 40% of the cereal production in SSA, where more than 80% is used as food, providing at least 30% of the total calorie intake to the people, with intake ranging from 52 to 450 g/person/day. In Latin America, maize consumption varies from 50 to 267 g/person/day (Poole et al. 2020). Since 1961, maize production worldwide has increased from 205 million metric tons to 1145 million metric tons (FAO 2020). This fivefold increase in maize production is primarily associated with increased yields in many regions of the world. However, in some regions, such as SSA, increased production is associated with a large increase in the area under maize production (187%), rather than maize yields, which only increased by threefold. The low productivity of maize in several maize-growing low- and middle-income countries, compared to global average of nearly 5 tons ha^−1^, could be attributed to various abiotic, biotic, and socioeconomic constraints (Shiferaw et al. 2011). In the tropics of SSA, Asia, and Latin America, maize is predominantly grown under rainfed conditions by resource-constrained smallholder farmers, often under the threat of devastating diseases and insect pests. Climate change adds further challenges to the existing problems and undermines the progress being made to improve the food security, incomes, and livelihoods of millions of maize-dependent smallholder farmers in the tropics.

Across the low- and middle-income countries, the population is projected to double by 2058 (UN World Population Prospects 2019). To meet the demands of the ever-increasing population, maize production must increase by 2.2% per year (Foley et al. 2011). Between 1981 and 2008 maize production was estimated to have increased annually by 1.7–1.8% worldwide; however, this figure masks significant regional variation (Iizumi et al. 2018). While production increased in 64–70% of maize-growing regions, there was either little improvement or even a decline in 21–23% of maize-growing area (Iizumi et al. 2018). Climate variability accounts for approximately one-third of the observed yield variability (Ray et al. 2015). For instance, the proportion of the yield variability attributable to annual climate variability was high in Zimbabwe (77%), Kenya (75%), Malawi (66%), Angola (61%), and South Africa (50%) (Ray et al. 2015).Over the last three decades, climate variability has resulted in a reduction in global maize production by 0.17 MMT per year, translating to an annual 0.7% decrease in consumable food calories available from maize globally (Ray et al. 2019). Climate change is further projected to reduce maize yields by an average of 7.4% for every 1 °C increase in mean global temperature (Zhao et al. 2017). Maize yield losses from insect pests are estimated to increase by 10–25% for every 1 °C warming through increased population growth and metabolic rates (Deutsch et al. 2018).

Current maize yield growth rate combined with population growth and predicted impacts of climate change will not suffice to meet future food demand. Improving crop productivity and livelihoods of smallholders under increasing climate variability will require a multi-disciplinary approach towards crop genetic improvement (Hansen et al. 2019). In terms of resource allocation, temperate maize has received much more resource over time compared to tropical maize (Andorf et al. 2019). However, the potential to directly impact smallholder farmers’ livelihoods through improved maize yields is higher in tropical environments. A recent study showed that gains in maize breeding have benefitted an estimated 53 million people in SSA (Cairns and Prasanna 2018). Increasing genetic gain, including a reduction in breeding cycle time, is key for providing farmers with a steady stream of improved varieties (Atlin et al. 2017; Andorf et al. 2019; Bailey-Serres et al. 2019).

Unlike in the temperate regions where the private sector maize breeding has been the major driver of yield gains, the public sector, including the Consultative Group of International Agricultural Research (CGIAR) centres, and their partners remain key players in genetic improvement in tropical environments (Renkow and Byerlee 2010; Hansen et al. 2019). Here we present a historical overview of CIMMYT’s intensive efforts over the last four decades, together with public and private sector partners in SSA, Asia, and Latin America, to develop and deploy climate-resilient improved maize germplasm. We will also highlight how modern tools and technologies, including high-throughput and precise field-based phenotyping, doubled haploid (DH) technology, genomics-assisted breeding, and breeding data management, and decision support tools, are enabling increases in genetic gains and improving the breeding efficiency of tropical maize breeding programs.

## Climate-induced stresses on tropical maize

Erratic precipitation and increase in temperature due to climate change have been projected to have the greatest effect on maize production and productivity, especially in SSA, Asia, and Latin America (Lobell et al. 2011; Smale et al. 2011), rendering the smallholder farmers particularly vulnerable (Cairns et al. 2012). While drought negatively affects all stages of maize growth and production, the reproductive stage, particularly between tassel emergence and early grain-filling, is the most sensitive to drought stress. Extreme sensitivity is mostly confined to the period − 2 to 22 days after anthesis, with a peak at 7 days, and almost complete barrenness can occur in drought-vulnerable cultivars. Drought stress during this period, resulting in a significant reduction in grain yield, is generally attributed to the separation of male and female flowering organs in the maize plant, and consequent effects on male–female flowering synchronization, and reduction in grain setting and kernel size (Bolaños et al. 1993). When drought stress occurs at the flowering stage, it is too late for the farmers to adjust management practices, and the season is too far advanced to consider replanting.

Heat stress alone, and in combination with drought, is becoming another major constraint to maize production (Cairns et al. 2013a). Temperatures are projected to increase by at least 1 °C, depending on the emissions scenario (Zhai et al. 2020). An increase in temperature of 2 °C would result in a greater reduction in maize yields than a decrease in precipitation of 20% (Lobell and Burke 2010). Frequent spells of high temperatures (often above 35 °C) along with moisture stress are a common phenomenon in most of the tropical semi-arid maize-growing regions, especially in South and South-East Asia, affecting especially the reproductive growth of maize. Unless frequently irrigated for maintaining high humidity (which may not be a possibility for many farmers in the tropics) to offset the effect of physiological drought, crop plants like maize will face compounded effects of heat and drought stress, leading to high yield losses.

Furthermore, the current trend of growing maize during the winter season, especially in the Indo-Gangetic Plains of South Asia, has increased the likelihood of maize crops exposed to suboptimal temperatures. The average minimum temperature in the winter season, especially in the North-West Plains of the Indo-Gangetic Plains, is below 5 °C. Besides early development, the flowering stage of maize crop is particularly vulnerable to chilling temperatures as this results in male sterility (Heslop-Harrison 1961) or very poor anthesis, and consequently, poor grain set (Thakur et al. 2010; Enders et al. 2019). Extreme cold stress was experienced by maize crops in northern India and the *Tarai* region of Nepal during the winter seasons of 2002–2003, 2009–2010, and again during 2017–2018, causing severe yield losses (Enders et al. 2019).

Waterlogging frequently affects more than 18% of the total maize production area in South and South-East Asia, causing production losses of 25–30% annually (Zaidi et al. 2010; Cairns et al. 2012). Soil waterlogging is a recurrent phenomenon wherever rainfall is erratic and intense, and the soil drainage capacity is poor. Because maize is a non-wetland crop species of tropical origin, it is highly susceptible to waterlogging at almost all the crop stages, especially before tassel emergence (Zaidi et al. 2004; Kuang et al. 2012).

In many regions of the world where maize is a major crop, an increase in soil salinity and/or irrigation water is one of the factors affecting maize production (Bänziger and Araus 2007). In general, maize is considered moderately sensitive to salt (Zörba et al. 2004; Chinnusamy et al. 2005), a category which comprises plants that maintain growth in saline soils with an electrical conductivity between 3 and 6 dS m^−1^ (Hasanuzzaman et al. 2013). Salinity stress can significantly affect maize seed germination (Munns and James 2003), vegetative growth (Szalai and Janda 2009), and reproductive capacity (Abdullah et al. 2001; Kaya et al. 2013). Excessive build-up of sodium and chloride ions in the rhizosphere leads to severe nutritional imbalances in maize due to strong interference of these ions with other essential mineral elements (Hasegawa and Bressan 2000; Karimi et al. 2005; Turan et al. 2010).

Climate models predict that extreme weather conditions, with more frequent droughts, higher temperatures, and heavy/erratic rainfall will also trigger greater intensity of diseases and insect pests, thereby imposing severe risks of crop failure. All the important life cycle stages of fungal, bacterial, and viral pathogens are directly influenced by environmental conditions like temperature, precipitation, humidity, and wind. Maize ear rots and stalk rots are groups of diseases that are generally found to have an increasing impact in changing climates. In Latin America, Asia, and SSA, changing environmental conditions, including high temperatures, drought, and soil nutrient deficiency might result in a shift in the distribution range and intensity of mycotoxin-causing fungi, such as *Fusarium graminearum* (causing *Gibberella* stalk rot, and *Gibberella* ear rot), *Fusarium verticillioides* (causing Fusarium stalk rot and Fusarium ear rot), and *Aspergillus flavus* (causing Aspergillus ear rot), hence the types of mycotoxins produced by these pathogens (De La Campa et al. 2005; Savary et al. 2011). Charcoal rot caused by *Macrophomina phaseolina* is another stalk rot that is highly impacted by hot and dry maize-growing seasons, prevalent under the conditions of high soil temperature of 30–42 °C, low soil moisture, and low soil pH (5.4–6.0) (Kumar and Shekhar 2005).

## Genetic architecture of key climate resilient traits in tropical maize germplasm

### Drought tolerance

Drought tolerance (DT) is one of the most complex quantitative traits in crop plants to study, characterize, and improve. The genetics of drought tolerance in maize has been extensively undertaken over the last three decades, especially for traits such as grain yield, and secondary traits, such as anthesis-silking interval (ASI), with strong genetic correlation with grain yield (Edmeades et al. 2017). Increased flowering synchrony (e.g. reduced ASI) was found to be strongly correlated to grain yield (Bolaños et al. 1993) and has since been extensively used in conventional breeding for drought tolerance. Since the 1990s, several publications have come out on molecular markers-based analysis of drought stress tolerance in maize, including various secondary traits associated with grain yield under drought-stressed environments in the tropics.

Ribaut et al. (1997) identified several small-to-moderate effect QTL associated with grain yield under different levels of drought stress; however, these QTL, in general, were not stable across different drought environments. Using a larger population and higher marker density, Messmer et al. (2009) identified six QTLs associated with grain yield under optimal and drought environments, with limited overlap of genomic regions identified across environments. Almeida et al. (2013) identified 83 QTL associated with yield under drought stress; each QTL explained 2.6 to 17.8% of the phenotypic variance. Seven meta-QTLs (mQTL) were identified across three populations, with six mQTL expressed under drought and optimal conditions. A meta-analysis of 18 bi-parental populations evaluated under a range of drought and optimal environments revealed 15 mQTL associated with grain yield (Semagn et al. 2013). However, mQTLs were not stable across environments and genetic backgrounds. Genome-wide association mapping studies (GWASs) on grain yield under drought, heat and optimal conditions identified several single nucleotide polymorphisms (SNPs) and candidate genes across locations; however, no overlapping SNPs were observed across treatments (Yuan et al. 2019). Thus, the lack of consistent and major phenotypic effects of the QTL in diverse recipient genetic backgrounds suggests that QTL-based marker-assisted selection is unlikely to play a major role in breeding for DT in maize.

### Heat tolerance

The major effect of heat stress on maize is reduced grain yield due to the impairment of photosynthetic and reproductive machinery. Apart from reduction in grain yield, high-temperature stress causes an overall reduction in growth, leaf scorching, reduced flowering time, increased ASI, and low pollen viability (Zaidi et al. 2016; Alam et al. 2017; Hussain et al. 2019; Jewel et al. 2019; Vinayan et al. 2019). Heat stress has not been found to delay silking in comparison with anthesis, although there could be a delay in silk receptivity due to heat stress. The major effect of heat stress on grain yield is primarily through reduced pollen viability, which in turn affects kernel number and grain set (Lizaso et al. 2018).

Molecular and physiological effects of heat stress have been investigated initially under controlled environments in maize seedlings and later under field conditions through managed heat stress phenotyping (Cairns et al. 2013b; Rattalino-Edreira and Otegui 2013). Frey et al. (2016) developed a heat susceptibility index for characterizing segregating families of temperate maize populations developed for heat stress tolerance characterization and identified two QTL hotspots on chromosomes 2 and 3 for various heat stress-associated traits. QTLs identified on chromosome 3 were found to be co-localized to the region previously identified for pollen viability under heat stress (Frova and SariGorla 1994). QTL hotspots for heat susceptibility index estimated for leaf scorching and plant height under heat stress have also been reported on chromosome 9 (Inghelandt et al. 2019).

CIMMYT, along with Purdue University, USA, and national agricultural research system (NARS) partners from South Asia, conducted GWAS on testcrosses of more than 500 diverse maize lines for grain yield and associated secondary traits under heat stress. The study identified five haplotype blocks and eight SNP variants for grain yield under heat stress. Twenty-two genomic regions identified through GWAS for grain yield or associated secondary traits under heat stress were validated by the CIMMYT team in Asia in independent biparental populations. Three major QTL intervals have been identified, based on the analysis of multiple populations, influencing various heat stress-related traits. CIMMYT’s GWAS panel was also studied for lipid traits along with field traits in heat-stressed and non-stressed environments, resulting in the identification of 78 significant SNP associations across 40 genetic loci with 53 candidate genes. In summary, recent studies undertaken by CIMMYT with partners in South Asia and the USA indicate that grain yield under heat stress or drought combined with heat stress in maize is polygenic in nature, and whole-genome prediction could be the most appropriate breeding method to improve yield performance under these stress conditions.

### Waterlogging tolerance

Genetic variation in maize for response to waterlogging was documented in temperate maize (Sachs et al. 1996), leading to an understanding that the inheritance and expression of traits associated with waterlogging tolerance in maize seedlings are physiologically and genetically complex (Subbaiah and Sachs 2003). Complicated responses to waterlogging, such as anaerobic proteins synthesis, alterations of gene expression, metabolic (switch to a fermentative pathway), and structural changes (e.g. aerenchyma formation), have been observed. Polygenic inheritance of waterlogging stress tolerance with partial dominance of tolerance over susceptibility was also inferred from a CIMMYT study involving tropical maize lines exposed to waterlogging at V7–V8 stage for 7 days (Zaidi et al. 2010). The study observed a predominance of additive variance over non-additive variance controlling grain yield under waterlogging.

QTL analysis of waterlogging tolerance using F2 families of a biparental population identified several QTL ranging from low to large effects, accounting for 3.9 to 37.3% of phenotypic variance (Qiu et al. 2007). Several QTL associated with shoot dry weight, root dry weight, total dry weight, plant height and their waterlogging tolerance coefficient were mapped on chromosomes 4 and 9 (Qiu et al. 2007). Waterlogging tolerance in teosinte, the wild progenitor of maize, showed some large-effect QTLs, especially affecting morphological modifications associated with the tolerance mechanism, like the constitutive aerenchyma formation and adventitious root development (Mano et al. 2016). Zaidi et al. (2015) studied a recombinant inbred line (RIL) population formed from waterlogging tolerant and elite susceptible lines from CIMMYT for grain yield and secondary traits under waterlogging stress in lines per se and test crosses at the V7-V8 stage. A total of six QTLs on chromosomes 1, 2, 3, 5, 7, and 10 were identified using RIL testcross dataset for grain yield under waterlogging stress, together explaining around 40% of phenotypic variance. The grain yield QTL on chromosome 1 and brace root QTL on chromosome 7 co-localized with previously identified constitutive QTLs for aerenchyma formation (Mano et al. 2010) contributed by teosinte accession, *Zea nicaraguensis*. Considering the polygenic nature of the trait, whole-genome prediction could be the most appropriate breeding method to improve yield performance under waterlogging stress.

### Cold tolerance

Cold tolerance in maize is reported to be of polygenic inheritance involving additive as well as dominance and maternal effects (Mahajan et al. 1993; Revilla et al. 2000). Several studies have been undertaken over the last two decades on cold tolerance, especially in temperate maize, identifying an array of putative QTLs distributed over maize genome influencing traits such as germination ability (Li et al. 2018), early seedling vigour (e.g. Hund et al. 2005; Rodríguez et al. 2014), early growth, and chlorophyll fluorescence (Strigens et al. 2013).

While most of the studies describe chilling tolerance at the seedling stage, little work has been done for cold stress during the vegetative or flowering stage. GWAS undertaken by CIMMYT on a set of 306 tropical testcross hybrids, including phenotypic evaluation under field conditions at three growth stages, viz. seedling, vegetative and pre-flowering/flowering stages, revealed 29 significant SNP associations common for the three stages associated with cold stress tolerance. A large of number of significant SNPs were found to be clustering at chromosomal bins 2.08, 6.01, and 10.04. These regions had also been previously identified for harbouring cold stress-associated traits (Fracheboud et al. 2004; Hund et al. 2004; Strigens et al. 2013; Hu et al. 2017). Nevertheless, considering the small effects of these QTL, marker-assisted selection is unlikely to be successful; whole-genome prediction is, therefore, suggested for improving cold stress tolerance in tropical maize.

### Resistance to diseases accentuated by the changing climates

Maize ear rots and stalk rots are groups of diseases that are generally found to have an increasing impact in changing climates. The inheritance of resistance to *Fusarium *spp. causing ear rots is complex. In diallel mating studies, hybrids were found to have 27% less ear rot and 30% less fumonisin content than their inbred parents (Hung and Holland 2012). Inbred performance per se and the corresponding general combining ability (GCA) in hybrids were significantly correlated. Though the phenotypic correlation between the severity of Fusarium ear rot and the amount of fumonisin was reported to be moderate to low (Clements et al. 2007), the genotypic correlation between the two traits was higher than the phenotypic correlation (Robertson et al. 2006). Several elite lines from CIMMYT with low accumulation of fumonisin content have been reported (Rose et al. 2017). QTL mapping studies in maize indicated that resistance to Fusarium ear rot is a quantitative trait determined by polygenes having small effects (e.g. Maschietto et al. 2017); however, a few studies detected QTL with moderate effects on chromosomes 3 and 4 (Ding et al. 2008; Chen et al. 2012a, b). GWAS identified SNPs associated with Fusarium ear rot and fumonisin content (Zila et al. 2013; Maschietto et al. 2017), some of which were located close to QTLs detected in biparental populations.

Many sources of resistance to *Aspergillus flavus* (*A. flavus*), including Mp313E, SC54, Mp420, and Tex6, were identified in temperate maize germplasm (Scott and Zummo 1988; Hamblin and White 2007), but resistance in the majority of them was highly environment-dependent. New breeding lines with repeatable and stable resistance were identified, majority of them with tropical germplasm background, especially using the Tuxpeño landrace (Warburton and Williams 2014). Resistance to *A. flavus* was found to be highly quantitative and inherited in an additive manner. Epistatic, dominant, and reciprocal effects were also reported in some studies (e.g. Williams et al. 2008). Many QTLs were identified with small effects, whereas some studies identified a few moderate to major effect QTLs that map to similar locations in different populations (Warburton and Williams 2014). A meta-QTL study conducted based on multiple QTL mapping experiments identified meta-QTLs with high confidence, a notable one on chromosome 4, which was significant across four different studies and three independent resistance sources (Mideros et al. 2014). There have been efforts to validate and transfer moderate to large effect stable QTLs for aflatoxin resistance across multiple genetic backgrounds by developing near-isogenic lines (NILs). Phenotypic trials of such NILs indicated that transfer of some QTLs could be highly effective. QTL pyramiding and reciprocal recurrent selection using marker-assisted recurrent selection (MARS) have been suggested for developing new resistant lines with stable resistance to *A. flavus* (Warburton et al. 2013).

Farrar and Davis (1991) and Henry et al. (2009) observed that maize genotypes behaved very similarly to *A. flavus* and *F. verticillioides*. Their data clearly showed high correlations between ear rot severities of the two pathogens and between aflatoxin and fumonisin concentrations. A meta-analysis including six of the QTL studies for *A. flavus* infection and six additional QTL studies for *Gibberella* stalk rot and Fusarium ear rot showed overlaps of resistance QTL to different fungal ear rots and identification of 62 meta-QTL on all chromosomes, except 9 and 10.

Post-flowering stalk rots are complex diseases, due to probable combined infection with multiple pathogens, accentuated by abiotic stresses and further compounded by secondary infections. Losses due to the stalk rots come in several different forms, including stalk breakage, lodging, premature death of the plant, and the interruption of normal grain filling process (Munkvold and White 1999). The degree of stalk rot infection depends greatly on environmental factors, genotype x environment interaction (GEI), and the resistance of given maize genotypes to the pathogens (Ledencan et al. 2003; Szőke et al. 2007). Resistance to different stalk rots is generally highly polygenic in nature, and in case of all stalk rots (Fusarium ear rot, *Gibberella* stalk rot, and Charcoal rotR) related to abiotic stresses, additive effects were found to be more significant than non-additive effects (e.g. Mir et al. 2018).

Genetic mapping studies for resistance to *Gibberella* stalk rot identified two QTL, namely *qRfg1* and *qRfg2*, increasing the resistance percentage of maize plants by 32–43% and ~ 125; these were fine-mapped to ~ 500 kb region on chromosome 10 and ~ 300 kb region on chromosomes 1 (Zhang et al. 2012). Functional genomic studies showed that *qRfg1* conferred its resistance through both constitutive and induced high expression of defence-related genes, while *qRfg2* enhanced resistance to the disease via relatively lower induction of auxin signalling and repression of polar auxin transport (Liu et al. 2016). Another QTL *Rgsr8.1* was fine-mapped, conferring broad-spectrum resistance to *Gibberella* stalk rot on chromosome 8 (Chen et al. 2017). GWAS in a panel of Asia-adapted CIMMYT maize lines led to identification of several SNPs across chromosomes associated with Fusarium ear rot and Charcoal rot. Two mapping populations were developed with one common susceptible parent for each of these stalk rots. These studies validated one QTL on chromosome 8 for Fusarium ear rot resistance, identified from GWAS. Similarly, two QTLs on chromosomes 6 and 8 identified in GWAS for charcoal rot resistance were also validated in biparental mapping populations.

## Breeding for climate-resilience in tropical maize germplasm

**Fig. 1:**
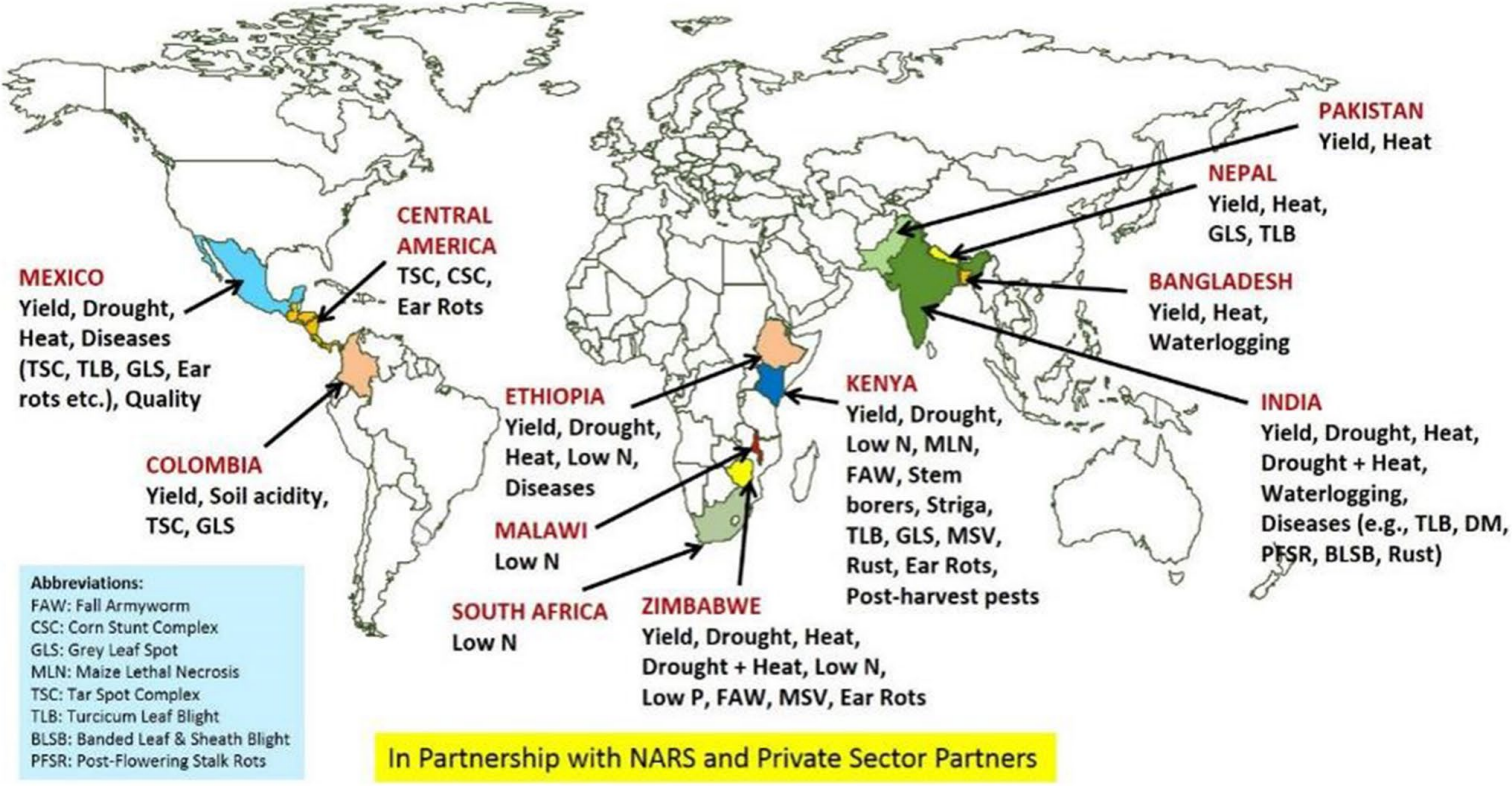
Maize germplasm phenotyping/testing network of CIMMYT and partners in the tropics of ESA, Latin America, and Asia

**Fig. 2:**
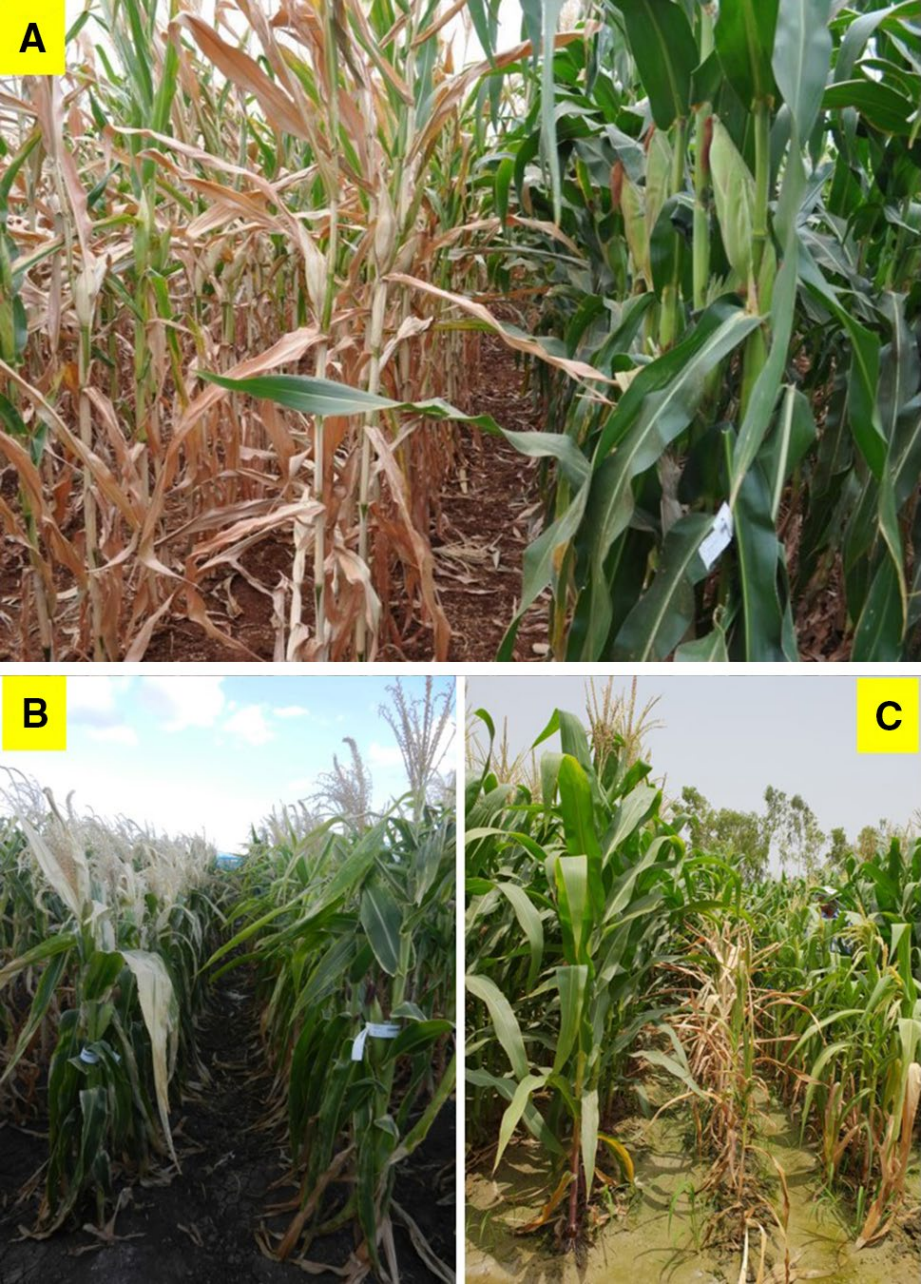
Phenotypic contrast of maize hybrids under managed drought stress (**a**), managed heat stress (**b**), and managed waterlogging stress (**c**) screening

CIMMYT’s breeding efforts for developing and deploying improved maize germplasm with climate resilience and other client-preferred traits are more than four decades old (Cairns and Prasanna 2018). CIMMYT follows a decentralized maize breeding strategy in SSA, Asia, and Latin America to reduce the effects of large GEI (Prasanna et al. 2020a). An extensive maize germplasm phenotyping/testing network in the tropics of SSA, Latin America, and Asia (Fig. 1) is at the heart of CIMMYT’s breeding strategy for developing multiple stress-tolerant maize varieties (Fig. 2). The breeding scheme was designed to develop products that perform well under both optimal and stressed environments (Cairns and Prasanna 2018). CIMMYT’s maize product advancement process typically includes not only regional on-station trials of promising pre-commercial hybrids coming out of the breeding pipeline vis-à-vis internal genetic gain checks and commercial checks but also extensive regional on-farm variety trials to ascertain the performance of the promising pre-commercial hybrids under farmer-managed conditions. This also provides an opportunity for the socioeconomic team to assess farmers’ own products as well as their trait preferences. The best entries coming out of this rigorous process are then allocated to public/private sector partners for varietal registration, scale-up, and delivery in the target geographies. This strategy has proved highly successful in developing and deploying impactful products, including a diverse array of improved maize hybrids and open-pollinated varieties (OPVs) adapted to the target markets (Beyene et al. 2017a, b; Cairns and Prasanna 2018; Worku et al. 2020).

Here, we briefly recount the historical efforts of CIMMYT in breeding climate-resilient maize germplasm for the tropics. In the mid- to late-1970s, CIMMYT began intensive efforts for developing improved maize germplasm with tolerance to drought stress (Fischer et al. 1983; Bänziger et al. 1997; Edmeades et al. 1997, 2017). The program started with recurrent selection for drought tolerance in the tropical white dent population Tuxpeño Sequíain 1975. At least eight cycles of full-sib recurrent selection under drought stress were completed at Tlaltizapán, Morelos, Mexico, where the rain-free period between November and April allowed for precise timing and intensity of stress levels. Recurrent selection programs started in other populations, including the Drought-Tolerant Population (DTP), La Posta Sequía, and Pool 26 Sequía, using S_1_ recurrent selection in the 1980s. The selection strategy in the 1980s involved pre-screening of large numbers of S1 families (500 to 600) under heat and drought stress during summer under temperatures exceeding 37 °C under semi-desert conditions at the Obregon research station in Sonora, Mexico, and then evaluating the selected 35–40% under drought stress in Tlaltizapán. Although heat stress was not considered as a priority trait at that time, screening germplasm in Obregon is likely to have indirectly incorporated heat tolerance into these populations. Thus, the recurrent selection programs produced improved populations (Table 1), notably Tuxpeño Sequía, La Posta Sequía, and the DTP yellow and DTP white, that served as source germplasm when the program switched to hybrid breeding. DT lines extracted from these populations have been used as donor germplasm for drought, low N and heat stress tolerance in SSA, Asia, and Latin America. A series of pools and populations were also developed, followed by several cycles of recurrent selections and improvement for targeted traits, such as tolerance to drought, low-N, heat, waterlogging, and soil acidity, in SSA, Asia, and Latin America (Edmeades et al. 2017). Several of the first-cycle lines from the DT populations were good enough to be released as CIMMYT Maize Lines (CMLs). At least 25 CMLs developed in Africa, Latin America, and Asia were based on DT populations Tuxpeño Sequía, DTP yellow, DTP white, and La Posta Sequía.

**Table 1:** Description and breeding history of key source populations for drought tolerance developed at CIMMYT-Mexico

Population	Trait	Initiated as	Cycles of selection	Breeding Scheme	Grain/Maturity	Adaptation
Tuxpeño Sequía	Drought	Tuxpeño-1 C12 & Tuxpeño-1 C6	Up to 8	FS & S1	White, dent; late	Lowland tropics of West Africa, Central America, Mexico & Andean region
La Posta Sequía	Drought	Pop. 43 C6	5	S1	White, dent; late	Lowland tropics of West Africa, Central America, Mexico, Andean Region, South & SE Asia
Pool 26 Sequía	Drought	Pool 26 C12	3	S1	Yellow, dent; intermediate/late	Lowland tropics of Mexico & Andean region
Pool 18 Sequía	Drought	Pool 18 C15	4	S1	Yellow, dent; early	Lowland tropics of South and SE Asia
Pool 16 Sequía	Drought	Pool 16 C12	5	FS/S1/S2	White, dent; early	Lowland tropics of West Africa
DTPW	Drought	DTP1/DTP2	8	S1	White, dent; intermediate	Lowland tropics / subtropics of Mexico, Sub-Saharan Africa, South & SE Asia
DTPY	Drought	DTP1/DTP2	8	S1	Yellow, dent; intermediate	Lowland tropics / subtropics of Mexico, Sub-Saharan Africa, South & SE Asia

Adapted from Edmeades et al. (1997)

In eastern and southern Africa, maize germplasm developed at CIMMYT-Mexico with drought-, low N-, and heat-tolerance provided a highly valuable foundation to further improve the target traits, including introgression of other defensive traits demanded by smallholder farmers in the target regions. For instance, an array of multiple stress-tolerant CMLs were released between 2006 and 2019, including CMLs 536 to 544 and 571 to 572, and 587 to 592, based on breeding efforts in southern Africa. In an effort to further boost the yield potential while improving on stress tolerance, CIMMYT maize breeding program introduced and utilized a wide range of off-plant varietal protection (Off-PVP) temperate maize inbred lines from the USA. CML593 is one of the first set of elite materials released from southern Africa with temperate-tropical introgression.

In Kenya, breeding for drought and low soil N stress tolerance was initiated in 1998 with the start of the Africa Maize Stress Project (Banziger and Diallo 2004). The source germplasm used to initiate the program was a combination of drought and low N tolerant germplasm from CIMMYT, besides early maturing populations from the International Institute of Tropical Agriculture (IITA). Populations used to extract lines included La Posta Sequía C3, La Posta Sequía C7, Tuxpeño Sequía C6, P43-SR, P49-SR, P501, P502, P22-SR, P25, P590, EV7992, and EV8449-SR. Lines extracted from these populations were used to form several synthetics and populations of intermediate to late maturity. The characteristics and source germplasm of some of the most important stress-tolerant populations and synthetics developed in Kenya and released in East Africa were described by Makumbi et al. (2018). Over time, more lines from La Posta Sequía C7 and DTPWC9 were used to increase the frequency of alleles for drought tolerance in new breeding starts. More recently, DT germplasm from CIMMYT-Zimbabwe, acid-tolerant germplasm from CIMMYT-Colombia, tropical lines from CIMMYT-Mexico, and off-PVP lines from USDA (as a source for yield potential and standability) have been used to develop multiple stress-tolerant germplasm adapted to target population of environments in East Africa. Thus, using various selection approaches across diverse testing environments, many inbred lines with good combining ability for DT and other adaptive traits were identified, and several elite CMLs and improved maize hybrids/synthetics were released. Many of the new stress-tolerant maize lines have been recycled through conventional pedigree or DH to develop better DT donor lines with higher productivity. These new donor lines have been used to develop multiple stress-tolerant hybrids that have been deployed across SSA (Cairns and Prasanna 2018). Efforts have now expanded to evaluate most hybrid combinations under drought and heat stress, not only to identify superior hybrids, but also to estimate breeding values of the inbred parents for grain yield under drought stress.

In Asia, breeding for drought tolerance was started in 2008 with the introgression of DT white maize donors from CIMMYT-Zimbabwe and from CIMMYT-Mexico (both white and yellow kernel colours). These donors were crossed to elite CIMMYT-Asia lines primarily bred for yield potential and regional adaptation, and with resistance to diseases like downy mildews and Turcicum leaf blight. Many bi-parental crosses were made after an extensive evaluation of a Design II between Asian adapted lines crossed to donor (African & Mexican) lines. CMLs 444, 505, 509, 440, 540, 542, and VL057847 were used as donors to derive 11 Asia-adapted DT populations (AMDROUT1-AMDROUT4, AMDROUT5 × 6, MARS7-MARS12) in a genomic selection (GS) scheme to derive Cycle2 (c2). These populations were evaluated per se and also in testcrosses under drought and optimal conditions. A comparison of the efficacy of GS versus phenotype-only selection for the development of source germplasm for drought clearly indicated the advantage of GS (up to 43% over phenotype-only selection) in achieving higher genetic gain per year (Vivek et al. 2017). Subsequent derivation of inbred lines and testcross performances confirmed the worth of the improved source populations for use as donors, especially AMDROUT1 and AMDROUT2. In addition, several inbred lines were developed through recycling/pedigree breeding within heterotic group biparental crosses or narrow-based breeding synthetics of 6–10 inbred lines. Testcross data indicated that germplasm backgrounds containing Suwan1, Cateto, P28, Composite15, and DTP lines gave good levels of drought tolerance.

Heat stress is a relatively new trait added to the portfolio of the CIMMYT maize breeding program based on studies indicating heat stress alone, and in combination with drought, is likely to become major constraints to maize production in the region of maize-dependent countries (Cairns et al. 2013b). The CIMMYT-Mexico maize program began screening populations for heat and drought stress in the semi-desert conditions of the Obregon research station in Sonora, Mexico, in the mid-1980s (Edmeades et al. 1997). Systematic efforts to develop elite Asia-adapted, heat-tolerant maize cultivars were initiated by CIMMYT in 2012 under the Heat Tolerant Maize for Asia (HTMA) project, in partnership with national maize programs in Bangladesh, India, Nepal, and Pakistan, and 15 seed companies operating in South Asia. This collaboration has enabled establishment of an extensive heat-stress phenotyping network, comprising 28 sites in the four Asian countries. To identify potential sources for heat stress tolerance, a panel of over 500 lines was constituted, named as Heat Tolerant Association Mapping panel, involving lines from DT populations from Latin America, SSA, and Asia. The panel was testcrossed and evaluated across 23 locations under natural heat stress during the spring season, where planting time was managed in such a way that most part of reproductive phase is exposed to heat stress. Promising lines were identified with good levels of heat tolerance and used in developing series of biparental populations. Recently, a total of six multiparent synthetic populations were developed, three each in heterotic group A and B, and improved using rapid-cycle genomic selection (RCGS). DH lines were extracted from improved cycle (C3), testcrossed, and evaluated over 20 locations under heat stress. Selected DH lines with good GCA for heat stress tolerance have been used to derive new generation of heat-tolerant inbred lines and hybrids adapted to South Asia.

In the 1980s, the maize breeding program of the Centro Nacional de Pesquisa de Milho e Sorgo (CNPMS/Embrapa) initiated the development of a genetically broad-based maize composite with waterlogging tolerance by recombining 36 populations (Ferreira et al. 2007). A modified stratification method of phenotypic recurrent selection was used for the development of this composite. After 12 cycles of selection, a waterlogging-tolerant maize variety, BRS4154 (Saracura), was released commercially (Ferreira et al. 2007). A targeted breeding program for developing improved populations, inbred lines and hybrids with waterlogging tolerance was initiated by CIMMYT in the year 2000 in collaboration with the Indian national maize program. Responses of tropical maize to the waterlogging stress and key secondary traits for waterlogging tolerance were identified (Zaidi et al. 2004, 2007a, b). A panel of maize lines derived from populations, local varieties or landraces from waterlogging-prone agro-ecologies were identified, testcrossed, and evaluated across locations for vegetative stage (V5–V6 stage) waterlogging responses. Based on across-locations performance, two populations, one each in intermediate maturity (WLS04) and late maturity (WLCY), were constituted by involving selected lines with GCA for waterlogging tolerance. A series of lines were extracted from these populations, and some promising trait donors and hybrids have been identified. Two multiparent synthetic populations were developed for waterlogging tolerance, one each in CIMMYT heterotic Groups A and B, and further improved using RCGS (rapid-cycle genomic selection). DH lines were extracted from the improved cycle (C3), testcrossed, and evaluated across locations under managed waterlogging stress. Selected lines with good combining ability for waterlogging tolerance are being used to develop a new generation of trait donors as well as improved maize hybrids with waterlogging tolerance.

Maize has significant intraspecific genetic variation for salinity tolerance (Mansour et al. 2005; Haque et al. 2015). Giaveno et al. (2007) confirmed genetic variability among hybrids for germination under salt stress and concluded that traits like seedling weight, growth rate, and photochemical efficiency should be used to screen salt-tolerant maize hybrids under salt stress. Under the CGIAR Research Program on Maize, in partnership with the International Center for Biosaline Agriculture (ICBA), Dubai, CIMMYT evaluated testcross progenies of a panel of 305 tropical maize inbred lines under managed salinity stress (8.0 dS m^−1^) and found significant genotypic variability, in terms of grain yield under salinity stress, ranging from 0.68 to 4.56 tons ha^−1^. Soares et al. (2018) phenotyped three CIMMYT maize lines (CML421, CML448, and CML451) alongside the reference B73 genotype under both control and salt-stressed conditions. The study revealed significant genotype-specific salinity stress responses in maize, and some elements of the underlying stress-response mechanisms. CML448 tolerated the highest ion content in its leaves whilst still able to grow; however, it displayed reduced vigour under control conditions. CML451 avoided the accumulation of ions in its shoots and displayed relatively strong vigour. Lower concentrations of a potentially channel-regulating protein and a higher abundance of proteins acting in protein synthesis and possible ion compartmentation were recorded in CML451.

Several studies on cold tolerance have been conducted in the temperate and highland maize. However, limited information is available on cold tolerance in tropical maize grown during the winter season in SSA, Asia, or Latin America. Adaptation of maize to the winter season in the tropics requires cold tolerance, that is, good seedling growth without suffering from cold-induced injuries. There is considerable genotypic variability in maize for traits that influence growth and development of maize under cold stress (Zaidi et al. 2010). Studies undertaken at CIMMYT indicated that tropical gene pools developed for high altitudes could possess valuable breeding materials for cold tolerance. Under the CGIAR Research Program on Maize, in partnership with two state agriculture universities in India, testcross progenies of a panel of 306 tropical maize inbred lines were screened under natural cold stress for three years (2017–2019), which revealed significant genotypic variability among test entries for various traits associated with cold tolerance, including grain yield. However, no cold-tolerant maize hybrids/OPVs have been so far released in tropical environments.

Various breeding methods have been used for development of improved tropical maize inbred lines with superior breeding values for grain yield in stressed environments, with tolerance to an array of abiotic and biotic stresses. Pedigree breeding method has been used to develop elite stress-tolerant inbred lines at CIMMYT and IITA in the last four decades. An array of source populations for drought, heat, and waterlogging tolerance (Tables 1 and 2) have been developed by CIMMYT teams in Mexico and Asia. These populations and narrow-based breeding synthetics, along with several bi-parental pedigree-based populations, are being used for inbred/DH line development in SSA, Asia, and Latin America by maize breeders at CIMMYT as well as national programs. A total of 603 elite CMLs have been released by CIMMYT as international public goods. The information about these CMLs can be accessed at https://data.cimmyt.org/dataset.xhtml?persistentId=hdl:11529/10246. These elite inbred lines are adapted to the lowland tropical, subtropical, tropical mid-altitude, and highland maize production environments targeted by CIMMYT and the partner institutions and have been extensively used as parental lines of several high-yielding, climate-resilient and disease-resistant maize hybrids (e.g. Bänziger et al. 2006; Magorokosho et al. 2006; Beyene et al. 2017a, b; Worku et al. 2016; Makumbi et al. 2018) and synthetics (Magorokosho et al. 2006; Masuka et al. 2017a, b).

**Table 2:** Source populations for abiotic and biotic stress tolerance developed by CIMMYT in Asia

Trait	Source populations developed	Key donors used in the formation of source populations
Drought	AMDROUT1(DT-Tester)c1F2, AMDROUT2(Ac)c1F2, AMDROUT3, AMDROUT4, AMDROUT(5 × 6), MARS7 to MARS12, G16BNSEQ-C3, DTPY-C9	CML444, CML505, DTP, CAL1717, CML562, CML564, Suwan1, Cateto, P45, P24, G16Seq, DTPY, CLRCY044, CML451, CML440, CML540, CML542, and VL057847
Heat	HSBC, MPS1, MPS2, MPS3, MPS4, MPS5 and MPS6	CML579, CML563, CML580, CL-RCY44, G18Seq, P31, CAL181, CML565
Waterlogging	WLS, WLCY, MPS-A and MPS-B	CML563, CML578, ZL154365, CAL1735, CML565

An important collateral effect of increasing selection intensity is the possible loss of genetic variation. This is true for any selection program. Intentional efforts to maintain adequate useful genetic variation within source germplasm pools are, therefore, an important consideration for any breeding program. Genebanks offer potential untapped source of novel alleles for key climate-related stresses (Navarro et al. 2017). Previously, a major limitation in the exploitation of genetic resources for pre-breeding from genebanks was the ability to identify of useful diversity from landraces (Mascher et al. 2019). In the past decade, over 4400 maize landrace accessions in CIMMYT genebank at Mexico have been genotyped and phenotyped for testcross performance to uncover allelic diversity for traits like flowering time (Navarro et al. 2017). Pre-breeding is also being undertaken at CIMMYT-Mexico for selected traits, such as drought tolerance, heat tolerance, resistance to maize lethal necrosis (MLN) and tar spot complex, and blue maize (Gorjanc et al. 2016; Terry Molnar, personal communication).

## Monitoring genetic gain for key climate-resilient traits

Estimating genetic gain within a breeding program is important to monitor efficiency and increase accountability. While estimates of genetic gain in temperate maize are well documented (Duvick 2005; Ci et al. 2011; Chen et al. 2016), estimates in tropical crops are relatively limited (Cobb et al. 2019). The first published study of genetic trend analysis at CIMMYT was published by Cordova et al. (2007) for the Latin America lowland maize breeding program. Grain yield under optimal conditions was estimated to have increased by 279 kg ha^−1^ year^−1^ between 1994 and 2002. The high rate of genetic gain in grain yield was attributed to the relatively young age of the program (less than 15 years). Over the past 10 years, there have been several studies on genetic gain in maize breeding programs at CIMMYT and IITA, especially in SSA (Table 3).

**Table 3:** Estimates of genetic gain in grain yield within tropical maize in advanced (Stage 4) breeding pipelines of CIMMYT and IITA in Sub-Saharan Africa.

	Genetic gain (kg ha^−1^ year^−1^)					
Breeding program	Optimum	Managed drought stress	Random/multiple stresses	Time period	Methodology	References
*West and Central Africa*						
OPVs, early maturity	30 (1.2)	14 (1.1)	40 (1.6)	1988–2010	Era study	Badu-Apraku et al. (2014)
OPVs, extra-early maturity	67 (2.3)	34 (3.3)	44 (2.7)	1988–2010	Era study	Badu-Apraku et al. (2015)
*Eastern and Southern Africa*						
Hybrids	109.4 (1.4)	32.5 (0.85)	22.7 (0.85)	2000–2010	Era study	Masuka et al. (2017a)
OPVs, early maturity	109.9 (1.76)	ns	29.2 (1.21)	2000–2010	Era study	Masuka et al. (2017b)
OPVs, intermediate maturity	79.1 (1.35)	ns	42.3 (2.09)	2000–2010	Era study	Masuka et al. (2017b)
*Southern Africa*						
Hybrids, early maturity	83 (1.81)	54 (2.12)	54 (1.83)	1999–2016	Era study	
Hybrids, early maturity	181 (2.2)	138 (2.0)	104 (1.9)	2013–2018	Rolling checks	
Hybrids, intermediate maturity	177 (1.9)	384 (2.5)	124 (2.5)	2013–2018	Rolling checks	
*Eastern Africa*						
Hybrids, intermediate maturity	130.7 (1.75)	79.3 (2.57)		2013–2017	Era study	
Inbred lines	39.3 (1.4)			1996–2013	Era study	Worku et al. (2016)

^*^Number in brackets is the rate of genetic gain (expressed in percent).

Through an era study In ESA, Masuka et al. (2017a) provided a baseline for genetic gains in grain yield across environments. Between 2000 and 2010, genetic gain for grain yield in the hybrid breeding program under optimal conditions, managed drought, random drought, and low N was estimated to have increased by 109.4 kg ha^−1^ yr^−1^ under optimal conditions, 32.5 kg ha^−1^ yr^−1^ under managed drought, 22.7 kg ha^−1^ yr^−1^ under random drought, and 20.9 kg ha^−1^ yr^−1^ under low N. A subsequent study on intermediate-maturity CIMMYT maize hybrids from eastern Africa released between 2013 and 2017 estimated genetic gain for grain yield optimal conditions at 130.7 kg ha^−1^ yr^−1^ and 79.3 kg ha^−1^ yr^−1^ under managed drought. In southern Africa, a study of hybrids between 2000 and 2018 estimated genetic gain in grain yield under optimal conditions at 126.8 kg ha^−1^ yr^−1^, at 55 kg ha^−1^ yr^−1^under managed drought, at 57 kg ha^−1^ yr^−1^under random stress, and at 76.5 kg ha^−1^ yr^−1^under low N.

The above studies of genetic gain were mostly done though era studies, except for Cordova et al. (2007), whereby varieties released in different years are evaluated in common trials. This approach avoids differences in agronomic management or climate change confounding the genetic trend (Rutkoski 2019). However, era studies require not only significant investment but also do not allow real-time monitoring of genetic gain. For these reasons, large private sector companies (especially in the USA) focused more on using historical breeding data for routine monitoring of genetic gain. As public breeding programs implement a range of new technologies to increase selection intensity and accuracy and reduce cycle time, continuous monitoring of genetic gain becomes increasingly important (Lenaerts et al. 2019). The use of rolling checks (across years) enables routine measurement of genetic gain, provided there is genetic connectivity across years for these checks.

Using historical data in the southern Africa maize breeding pipeline, grain yield is estimated to have increased between 2013 and 2018 by 181 kg ha^−1^ year^−1^ under optimal conditions, 138 kg ha^−1^ year^−1^ under managed drought stress, 104 kg ha^−1^ year^−1^ under random stress, and 75 kg ha^−1^ year^−1^ under low N in the early maturity group. In the intermediate maturity group, grain yield is estimated to have increased between 2013 and 2018 by 177 kg ha^−1^ year^−1^ under optimal conditions, 384 kg ha^−1^ year^−1^ under managed drought stress and 124 kg ha^−1^ year^−1^ under random stress (Cairns et al. submitted). An important consideration in genetic trend analysis is the percentage of entries which are checks (check %). The number of checks and the checks themselves influence the estimate of genetic gain.

In the early days of maize hybrid development programs in SSA, the source germplasm which was available to the breeders for line development was largely unimproved and intolerant to inbreeding depression. Understanding the problem with seed production, breeders used early generation parental inbred lines (not fixed) and developed some commercial hybrids. However, these hybrids had challenges because of a lack of uniformity in seed production and farmers’ fields (Ertiro et al. 2015). CIMMYT and IITA established strong maize breeding programs in SSA over the last three decades and have developed several biotic and abiotic resilient productive fixed inbred lines. A recent study showed that the productivity of Africa-adapted CIMMYT maize lines has increased significantly over the years (Worku et al. 2016). For example, the mean grain yield for the inbred lines developed before 2000 was 2.7 tons ha^−1^, while it was 3.4 tons ha^−1^ for the inbred lines developed from 2011 to 2013. The top-yielding 10 inbred lines had mean grain yield of 4.3 tons ha^−1^, which is still low as compared to the temperate inbred lines developed over 100 years, 6.0 tons ha^−1^ (Duvick 2005). The mean grain yield improvement (genetic gain) of CIMMYT tropical maize inbred lines over 18 years was estimated to be 1.4% per year, or 39.3 kg ha^−1^ year^−1^ (Worku et al. 2016), indicating good progress over a relatively short period of time. However, there is significant scope for further improvement, as this genetic gain is less than that (2%) reported for temperate inbred lines (Troyer 2006).

## Increasing genetic gain in the stress-prone tropical environments

Even though the gains in grain yield in tropical maize are comparable to other regions of the world, absolute yield in farmers’ fields remains low (Cairns and Prasanna 2018). Genetic gain can be improved in several ways. Initial implementation of replicated trials across a large regional testing network has led to improved selection accuracy for key traits. Although effective, expansion of phenotyping capacity will, ultimately, always be limited. Increasing population size increases the probability of identifying superior progenies through greater selection intensity.

**Fig. 3:**
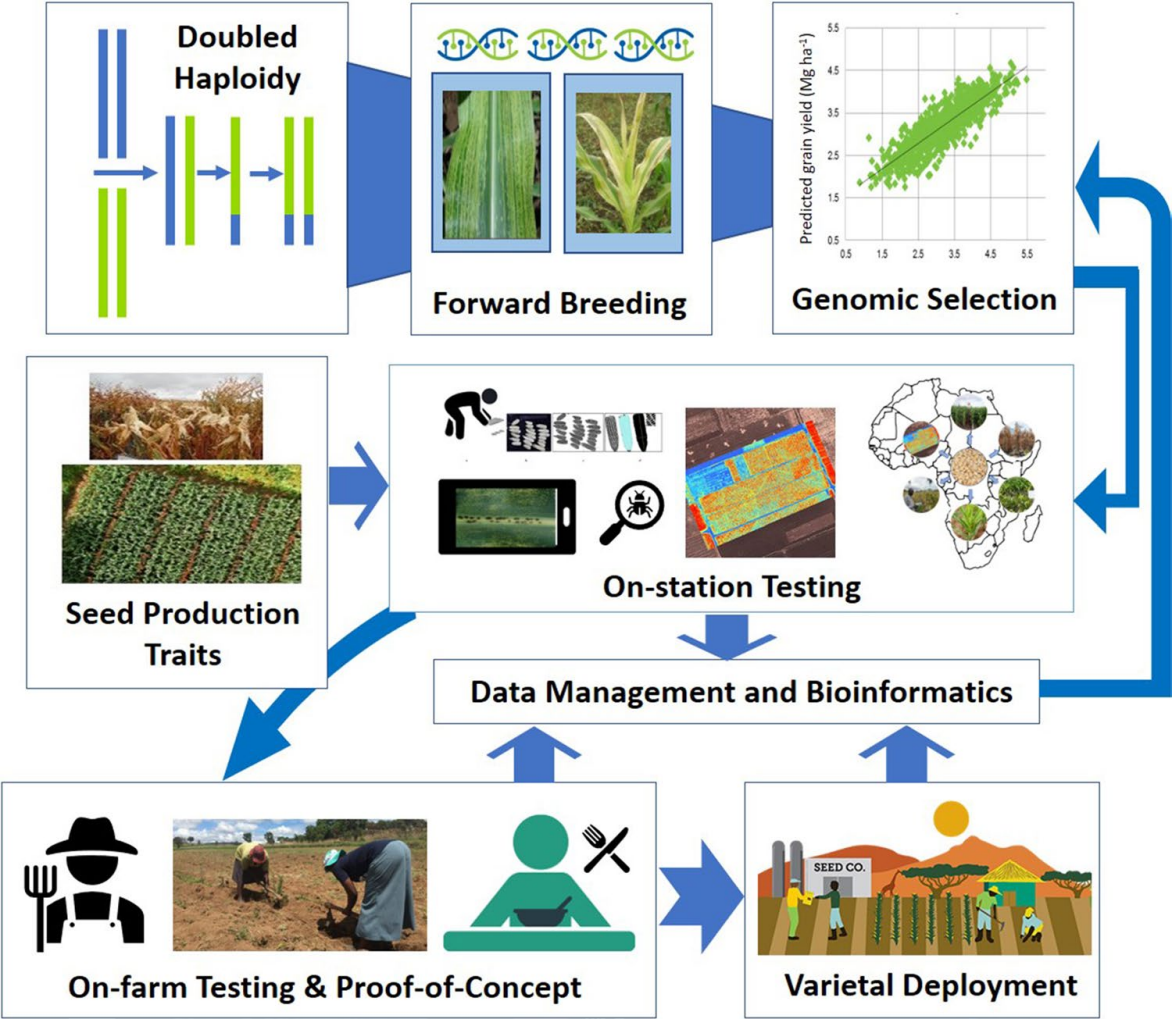
Schematic depiction of the maize breeding pipeline of CIMMYT for developing and deploying elite multiple stress-tolerant tropical maize germplasm for sub-Saharan Africa, Asia, and Latin America

If the accuracy and intensity of selection are constant, reducing breeding cycle time by half will double the rate of genetic gain (Atlin et al. 2017). The best private-sector programs take three to four years for one breeding cycle, whereas public sector breeding programs in developing countries usually have very long breeding cycles. A “breeding cycle” is here defined as the time from when a new breeding population is generated to when the best new lines derived from this population are used as new breeding parents (Atlin et al. 2017). If equivalent estimation of parental breeding value can be maintained while halving cycle time, the rate of genetic gain will be doubled. Reduction of cycle time has the added potential benefit of increasing the frequency with which haplotypes are recombined and exposed for selection in the constantly changing environment (Lenaerts et al. 2019). This increases the probability of creating and selecting allelic combinations that are closer to optimal for current conditions. Rapid breeding cycles are also critical for adaptation against evolving pest and pathogen populations. Although cycle time reduction is a high leverage intervention for improving genetic gain, a critical number of growing seasons for phenotypic evaluation of new products will always be essential as the year effect component of the genotype x environment interaction is substantial and robust new varieties need to be evaluated adequately in varying conditions. To increase genetic gain in tropical maize breeding, DH, forward breeding, GS, high-throughput phenotyping, and extensive on-station and on-farm testing are being deployed by CIMMYT across ESA, Latin America, and Asia (Fig. 3).

### High-throughput and precise field-based phenotyping

In crop improvement, accuracy of the phenotyping process is a significant contributor to genetic gain. In recent years, we have seen an unprecedented rise of interest in the development and use of high-throughput and precise phenotyping protocols by research institutes and private companies for recording various traits used in breeding programs. These are largely based on remote sensing and include the use of multispectral, hyperspectral, fluorescence, and thermal sensors. The development of field-based high-throughput phenotyping methods covers both ground-based (including handheld) and aerial sensing methods.

To support the development of climate-resilient varieties and through strategic partnership, the CIMMYT maize breeding program, has been developing and deploying various sensing-based tools/methods for improving phenotyping throughput and accuracy (Zaman-Allah et al. 2015; Makanza et al. 2018a). These tools/methods have made it possible to quantitatively measure the key traits relevant to drought or heat stress tolerance like early vigour, canopy senescence, with comparable or higher accuracy at a lower cost compared to visual assessments (Cerrudo et al. 2017; Makanza et al. 2018a). Similarly, digital imaging has been used to rapidly assess yield component traits that can provide insights about tolerance to abiotic stresses like drought or heat as well as tolerance to pests like Fall Armyworm (Makanza et al. 2018b). Limited options are currently available for disease or pest damage detection, spatial distribution, and evaluation. Canopy reflectance in visual and near-infrared wavebands has been used at CIMMYT-Mexico for tar spot complex phenotyping in maize (Loladze et al. 2019).

In addition to saving time, substantial cost reduction can be achieved by using high-throughput phenotyping for routine traits; this in turn enables an increase in selection intensity by increasing the number of plots that are effectively phenotyped (Araus et al. 2018). In the future, unmanned aerial vehicle (UAV)-based sensing is anticipated to replace the ground-based phenotyping methods. The integration of image processing and standardized analytical pipelines will likely enable non-experts to process and analyse the generated data. Given the rapid advancements in artificial intelligence (also referred as deep/machine learning), high-throughput field phenotyping tools will improve the heritability and accuracy of several traits that are often evaluated manually, generate more robust data pipelines, and facilitate the use of complex datasets in breeding decisions.

### Doubled haploid (DH)-based breeding

DH technology enables efficient and rapid production of completely homozygous inbred lines for maize breeding programs (Prasanna et al. 2012; Chaikam et al. 2019). Although DH does not reduce cycle time relative to early-generation testing schemes (testcrossing F3 families), using DH technology, homozygous lines can be developed in two crop seasons compared to six to eight seasons of inbreeding through the conventional method (Prasanna et al. 2012; Sleper and Bernardo 2016; Chaikam et al. 2019). As they are homozygous, DH lines enable greater selection efficiency. This, combined with higher variation between entries compared to early-generation testing methods, improves overall realized heritability, resulting in higher genetic gain. DH has been increasingly adopted in CIMMYT maize breeding programs, especially since 2010, gradually replacing pedigree breeding (Chaikam et al. 2019). Since 2013, CIMMYT has developed more than 200,000 maize DH lines from diverse source populations and successfully identified maize DH lines with superior characteristics for use in breeding pipelines in SSA, Latin America, and Asia. These DH lines have superior per se performance (Worku et al. 2016; Beyene et al. 2017a), good combining ability for stress tolerance (Beyene et al. 2013, 2017a, Erito et al. 2017; Sserumaga et al. 2018), and tolerance/resistance to various diseases (Beyene et al. 2017b).

Since 2013, CIMMYT has produced more than 250,000 DH lines from diverse Africa-adapted maize genetic backgrounds which have been used to develop several elite lines and hybrids. To date, seven DH lines have been released as CMLs (CML566, CML567, CML568, CML569, CML570, CML584, and CML603). More than 80 improved CIMMYT-derived maize hybrids involving DH lines as parents, and exhibiting superior performance under optimum, drought, and low nitrogen stress conditions have been released by national/regional partners in Kenya, Uganda, Tanzania, Mexico, and South Africa between 2012 and 2017 (Beyene et al. 2017b; CRP MAIZE Annual Report 2019).

### Genomics-assisted breeding

Molecular technologies offer the ability to expand the size of a breeding program, thereby increasing selection intensity, without increasing phenotyping requirements. Genotypic information can be used to select germplasm prior to the phenotyping stages, and the capability to increase this phenotypically untested layer will allow the total number of genotypes within a breeding program to be expanded (Cooper et al. 2014). When carefully implemented, molecular strategies can also reduce breeding cycle time by two to three years (Gilliham et al. 2017). Without increasing phenotyping requirements, genomics-assisted breeding can increase selection intensity and shorten the cycle time, thereby accelerating the genetic gain. Moreover, the genotyping cost per sample (for screening a few to thousands of markers) has decreased dramatically over the years, making it equivalent to or even cheaper than the cost of phenotyping a single plot of maize in multi-environment trials.

Several strategies on how to effectively use genotypic information to improve the efficiency of crop breeding programs have been devised, based on trait architecture, genotyping cost, and breeding objectives; these include quality assurance (QA)/quality control (QC), marker-assisted backcrossing (MABC), forward breeding, and GS. QA/QC focuses mainly on the genetic quality related issues of the improved seed. QA is used to prevent the low-quality or genetically impure seed to be mixed with high-quality seed, while QC in the seed production chain is used to ensures identification and correction of possible errors or mixtures that might have slipped through QA protocols. Molecular markers-based QA/QC analysis has been routinely implemented in CIMMYT maize breeding programs, especially in terms of testing for genetic purity and identity, parent-offspring test, and trait-specific testing using breeder-ready molecular markers (Gowda et al. 2017). MABC has been successfully used at CIMMYT to introgress resistance to maize lethal necrosis (MLN) into over 30 elite, Africa-adapted, DT but MLN-susceptible lines (Prasanna et al. 2020b). FB is a simple form of population enrichment using markers tightly linked to genomic regions of high importance. FB is being deployed in tropical maize breeding to enrich breeding populations for favourable alleles of large-effect genes or QTL for resistance to important diseases, such as maize streak virus (MSV), and MLN, besides nutritional quality traits, such as provitamin A (Prasanna et al. 2020c). GS, a widely adopted strategy in advanced crop breeding organizations, uses genome-wide marker information to estimate all marker effects and to select for individuals with high genomic-estimated breeding values (GEBVs) (Meuwissen et al. 2001; Santantonio et al. 2020).

CIMMYT has an active trait pipeline in maize with a series of six stages, including marker discovery, field validation, haplotype optimization in breeding parents and populations, and technical validation of genotyping assays, before deploying either through FB or MABC. Most of the traits in this pipeline are adaptive traits associated with key biotic stresses or nutritional quality (Table 4). The markers which are deployed are being routinely screened to enrich favourable alleles in selection stages in the breeding pipeline. In maize, traits associated with abiotic stress tolerance are largely complex due to polygenic inheritance and high GEI. The genetic complexity of most of the traits related to stress tolerance is compounded due to the multiple physiological and phenological mechanisms leading to tolerance, in terms of producing reasonable grain yield under stress conditions (Campos et al. 2004). In this situation where the effect size of the QTL in the breeding germplasm is not found to be sufficient enough to justify the cost of discovery, validation, and deployment of the locus, GS is being used to take into account all QTL, minor or major, spread across the genome (Meuwissen et al. 2001; Santantonio et al. 2020).

**Table 4:** Breeder-ready marker discovery-validation-deployment pipeline for some of the client-preferred/climate-adaptive traits in tropical maize germplasm. The black dots represent the present stage of the trait in the pipeline

Traits	Discovery	Field validation	Haplotype optimization		Assay verification	Deployment
			Breeding parents	Breeding Populations		
Maize streak virus (MSV)						●
Maize lethal necrosis (MLN)						●
Turcicum leaf blight (TLB)					●	●
Tar spot complex					●	
Grey leaf spot (GLS)					●	
Common rust		●				
Fusarium ear rot (SSA)		●				
Fusarium ear rot (Latin America)	●					
Post-flowering stalk rot (Asia)		●				
Fusarium stalk rot (Latin America)	●					
Aflatoxin (SSA)		●				●
Striga		●	●			
Fall armyworm	●					
Provitamin A						●
Kernel Zinc				●		

The effectiveness of GS is a product of the quality of the training population with both genotypic and phenotypic data used to estimate the marker effects in the predicted population. GS is especially useful for complex traits. CIMMYT breeding programs have evaluated several GS-related methods with varying levels of success over the past decade (e.g. Crossa et al. 2010; Windhausen et al. 2012; Burgueño et al. 2012; Beyene et al. 2015, 2019; Zhang et al. 2015, 2017a,b; Vélez Torres et al. 2018; Wang et al. 2020). Refinement in strategy coupled with the reduction in cost of securing adequate marker density has enabled the mainstreaming of GS as an integrated breeding method. Genomic prediction could be applied to source population improvement by way of rapid cycling and could lead to an improvement in genetic gains primarily due to changing allele frequencies through the use of markers in a time-efficient manner. In a study of rapid cycle genomic selection in eight biparental populations in eastern Africa, the average gain from GS per cycle across eight populations was 0.086 tons ha^–1^. The average grain yield of Cycle 3-derived hybrids was significantly higher than that of hybrids derived from Cycle 0. Hybrids derived from C3 produced 7.3% higher grain yield under drought than those developed through the conventional pedigree breeding method (Beyene et al. 2015). In two biparental RCGS for deriving improved stress-tolerant lines in the CIMMYT-Asia breeding program, a gain of 10–20% in grain yield under drought was observed after two cycles of GS, compared to phenotypic selection (Vivek et al. 2017).

RCGS is also applied in multi-parent synthetic populations in CIMMYT breeding programs to increase the efficiency of line derivation. In a multi-parent population, a 7.74% increase in genetic gains was observed from cycle 1 to cycle 4 under optimal conditions for grain yield (Zhang et al. 2017a,b). Analysis of two multi-parent populations improved through RCGS for drought and waterlogging stress tolerance in Asia showed a realized genetic gain under drought stress of 0.110 tons ha^−1^ year^−1^ for heterotic group A populations and 0.135 tons ha^−1^ year^−1^ for heterotic group B populations (Das et al. 2020). The observed gain was less under waterlogging stress, where heterotic group A population showed 0.038 tons ha^−1^ year^−1^ and heterotic group B population showed 0.113 tons ha^−1^ year^−1^. Genomic selection for drought and waterlogging tolerance resulted in no yield penalty under optimal moisture conditions (Das et al. 2020).

Apart from population improvement, genomic prediction based on early-stage yield testing (Stage 1) is an important tool in the modern maize breeding pipeline, enabling increased selection intensity and reduced cost and time. In a proof-of-concept study in a set of 22 biparental populations evaluated for grain yield and other agronomic traits, moderate to high prediction accuracies were obtained with higher heritability and with a training population size that was at least 50% of the total population (Zhang et al. 2017a). The strategy of test-half-and-predict-half based on marker data has been piloted in specific product profiles of CIMMYT in Africa and Latin America with highly encouraging results, while in Asia progress has been made to implement GS for heat and drought related traits. Beyene et al. (2019) compared GS versus phenotypic selection in CIMMYT maize breeding program in SSA and reported that there was no significant difference between the mean of hybrids advanced through phenotypic and GS both under optimum and managed drought stress conditions, but GS reduced the cost by 32% over phenotypic selection. This strategy of testing-half-and-predicting-the-remaining based on marker data should be effectively incorporated into maize breeding pipelines to enhance the efficiency of breeding programs. Wang et al. (2020) evaluated the prediction accuracy across years, when a historical multiple-year training dataset was used to predict the GEBVs of the untested lines in the subsequent years. The average observed prediction accuracy of grain yield was 0.32 when one-year historical data were used as training population to predict the other year data as testing population. The prediction accuracy increased to 0.42 by utilizing two-year historical data as training population. This preliminary result showed that the genomic prediction accuracy across years is equivalent to the phenotyping accuracy of the Stage 1 trials. GS with a multiple-years training data offers the opportunity to even eliminate Stage 1 testing, thereby reducing significantly the breeding cycle time and cost.

### Breeding data management and decision support tools

A breeding informatics system capable of handling all breeding operations is key to enhancing genetic gains and ensure the success of any plant breeding program (Cobb et al. 2019). Such a system should, at a minimum, help breeders to track the germplasm accurately at each stage in the pipeline, reduce manual handling of data, eliminate errors during germplasm handling by technical staff, improve efficiency of handling data with user-friendly software tools/program, undertake effective data analyses, and finally decision-making. The data management system “Fieldbook” (Bänziger and Vivek 2007; Vivek et al. 2007) has been used for more than two decades by CIMMYT and partners. However, recent advancements in genotyping, high-throughput phenotyping statistical designs, and their analyses, and predictive breeding algorithms mean that a basic breeding database is no longer sufficient to handle different types of information that modern breeding programs are required (Ye et al. 2013).

Phenotyping precision under stress can be improved by combining yield trial data, molecular data, and historical phenotypic and pedigree information using Best Linear Unbiased Predictors (BLUP). Predictive breeding can be especially rewarding in maize because it is relatively easy to produce more DH lines than breeders can test in the field. Predictive breeding techniques can be used to select those lines that cannot be tested in the field due to limited resources if enough historical phenotypic data and pedigree information are available. These capabilities are all essential, but for their potential to be fully realized, a breeding informatics system that integrates the necessary tools and data is equally indispensable. To address the increasing need to handle more complex types of data and to facilitate the sharing of data among breeding programs, CIMMYT and other CGIAR centres are developing an Enterprise Breeding System (EBS). The EBS promises to increase breeding efficiency through better decision support by integrating modern breeding tools into one system, enabling data sharing through improved interconnectivity, and allowing monitoring and evaluation of breeding programs.

### Capacity development and adoption of new tools and technologies

Effective integration of new tools and technologies into NARS breeding programs is essential for long-term sustainability in maize breeding (Cobb et al. 2019) and the capacity to develop climate-resilient cultivars with greater efficiency. Historically, training of NARS partners has been a significant component of CGIAR breeding. However, there is an increasing recognition of the need for more collaborative and targeted capacity development based on the current capacity of NARS partners. Coordinated breeding networks and joint product advancements are important for public breeding programs targeting similar agro-ecologies. The use of a coordinated breeding network allows partners access to specialized phenotyping facilities, besides access to a larger network of managed stress screening sites.

Unlike the private sector where top-down decisions are made regarding incorporation of new tools into breeding programs, public sector breeders have a relatively high degree of decision-making power. Moreover, adoption of new tools/technologies within a public sector institution is a function of several internal and external variables. Understanding the factors influencing the adoption of new tools/technologies and associated constraints to adoption, will help facilitate outscaling. In rice, a survey of adoption of new technologies in public sector breeding identified time-saving as the most important variable influencing willingness of breeders to adopt, followed by cost and labour savings (Lenaerts et al. 2018). Perceived genetic gain ranked relatively low on factors associated with willingness to adopt.

Relative to the wealth of information on new breeding tools/technologies/schemes presented in the current literature, cost–benefit analyses of those new tools/technologies are rarely analysed or presented (Dreher et al. 2003; Morris et al. 2003). Literature around the adoption of new technologies within public sector breeding programs and barriers to adoption is even scarcer (Lenaerts et al. 2018). Adoption of new technologies within the public sector in the current climate of funding uncertainties will be, in part, by value propositions for new technologies.

## Deployment of improved climate-resilient maize varieties in the tropics

CIMMYT, in partnership with national programs and the private sector, is intensively engaged in deploying improved climate-resilient maize varieties for tropical/subtropical environments in SSA, Asia, and Latin America. In ESA, climate-resilient maize varieties typically yield up to 20–25% more than benchmark commercial varieties in on-farm trials under low-input and drought stress conditions (Setimela et al. 2017). In 2015–2016 crop season, under the severe El Nino induced-drought and heat stress conditions in southern Africa, CIMMYT-derived drought-tolerant maize hybrids yielded twofold more than key commercial hybrids in on-farm trials (Setimela et al. 2018). Crop modelling shows climate-resilient varieties could provide a yield advantage of 5–25% in many maize-growing areas in ESA (Shiferaw et al. 2011).

**Fig. 4:**
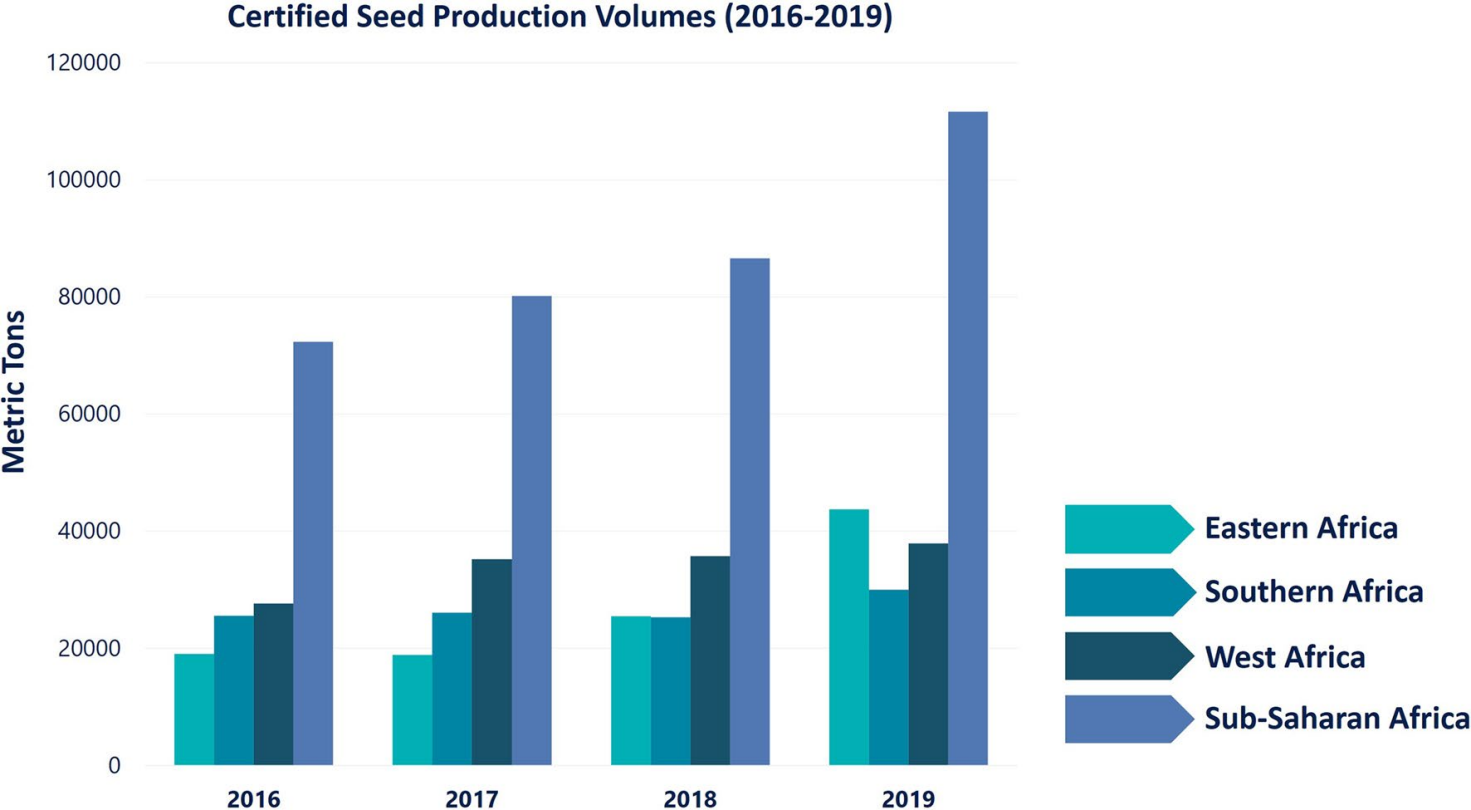
Certified seed production volumes (in MT) of CIMMYT/IITA-based improved stress-tolerant maize cultivars in eastern, southern, and West Africa, and overall, in sub-Saharan Africa in 2019

Under the Stress Tolerant Maize for Africa project, during 2016–2019, a total of 218 new stress-tolerant and productive improved maize varieties derived from CIMMYT and IITA germplasm were officially released (76 in 2016, 44 in 2017, 54 in 2018, and 44 in 2019) to enter commercialization, promotion and wide-scale dissemination in 13 target countries across SSA. By the end of 2019, the total certified seed production of stress-tolerant improved maize varieties reached 111,713 MT (Fig. 4), which is estimated to be adopted in 5.03 million hectares in eastern Africa (Ethiopia, Kenya, Tanzania, Uganda), southern Africa (Malawi, Mozambique, South Africa, Zambia, Zimbabwe), and West Africa (Benin, Ghana, Mali, Nigeria).

Since 2015 there have been several success stories of the replacement of old and climate-vulnerable maize varieties with newer stress-tolerant varieties in SSA. One such example from Ethiopia is the replacement of the hybrid BH660 (released in 1993) with a new climate-resilient hybrid BH661 (released in 2012). Certified seed production volumes of BH660 (released in 1993) dropped from 5778 MT in 2012 to less than 1300 MT in 2019, while commercial seed volume of BH661, a climate-resilient maize hybrid released in 2012, rose to 8,433 MT by 2019 (Ertiro et al. 2019). The remarkable success in terms of varietal releases, seed scaling, and wide-scale deployment of climate-resilient improved maize varieties in SSA has been accomplished in close partnerships with nearly 100 local/regional seed companies and national agricultural research organizations across 13 target countries. For instance, in 2019 more than 25 seed companies in Uganda, Kenya, and Ethiopia produced 40,657 certified seed of stress-tolerant maize varieties (purely CIMMYT germplasm-based and combination hybrids), on average which can cover 30% of total maize area in the three countries. CIMMYT and IITA supported the seed company partners, especially the small and medium enterprises, in the supply of early generation seed, and technical backstopping in terms of hybrid seed production, seed road maps, market segmentation, territory planning, seed business management, and varietal replacement.

In the South and South-East Asia, CIMMYT, in active collaboration with national programs and private sector seed company partners, has successfully implemented a series of regional collaborative projects focussing on developing climate-resilient maize germplasm with tolerance/resistance to an array of major abiotic and biotic stresses without compromising on yields under optimal conditions. These projects include Affordable, Accessible, Asian Drought-tolerant Maize (AAA), Heat Tolerant Maize for Asia (HTMA), Climate Resilient Maize for Asia (CRMA), Improved Maize for Tropical Asia (IMTA), and the International Maize Improvement Consortium for Asia (IMIC-Asia). The projects specifically focused on developing elite, high-yielding, and climate-resilient maize with tolerance to drought, heat and/or waterlogging, and also tolerance to a combination of stresses, besides resistance to major diseases. Since 2015, a total of 35 CIMMYT-derived improved maize cultivars (22 hybrids and 13 OPVs) with multiple stress tolerance have been released and commercialized in Asia. Seed scaling is now gaining momentum; in 2020, over 500 MT of stress-tolerant hybrids were produced by seed company partners. Further, CIMMYT in Asia is targeting marginalized rainfed agro-ecologies with little penetration of improved maize hybrids from large multinational companies. For instance, in Nepal and Pakistan, an estimated 1.0 million hectares of maize area is being cultivated under rainfed conditions by smallholder farmers with most of the commercially available or released maize varieties sourced from the CIMMYT maize breeding program. In 2019, Pakistan alone released 10 new CIMMYT-derived drought-tolerant maize varieties suitable for rainfed conditions. In Nepal over 60% of the maize area in the high-hills and mid-hills areas is rainfed. Three new CIMMYT maize varieties released in 2015 and 2018 in Nepal, serve as replacement for the old varieties like Rampur Composite (released in 1975) and Arun-2 (released in 1982).

In Latin America, CIMMYT, in active collaboration with national programs, non-government organizations and private seed companies, has been successfully implementing a regional collaborative testing network focussing on developing climate-resilient maize germplasm without compromising on yield under optimum conditions. During 2012–2019, under the Sustainable Modernization of Traditional Agriculture (MasAgro) project, a total of 70 new stress-tolerant and productive maize varieties derived from CIMMYT germplasm were officially released for wide-scale commercialization in Mexico. By the end of 2019, the total seed production of CIMMYT-derived stress-tolerant improved maize varieties reached 22,754 MT, which is estimated to be adopted on 1.14 million hectares in rainfed growing areas of Central, West, and South-east Mexico.

Further, CIMMYT in Latin America is targeting marginalized lowland (0–1000 m above sea level, masl) tropical rainfed agro-ecologies with little penetration of improved maize hybrids from large multinational companies. For instance, in Guatemala, Honduras, Colombia, and Venezuela, an estimated 1.1 million hectares of maize area is being cultivated under rainfed conditions by smallholder farmers with most of the commercially available or released maize varieties sourced from the CIMMYT maize breeding program. In the past two years, Guatemala and Colombia released seven new CIMMYT-derived maize varieties suitable for rainfed conditions. The new CIMMYT maize varieties released in Guatemala are expected to replace old varieties like HB-83, which was released in 1990. Extensive public–private partnerships through MasAgro and CRP MAIZE have supported a growing seed sector in Latin America and enabled many maize-based seed companies to expand/renew their product portfolios, replacing obsolete hybrids/varieties with CIMMYT-derived improved stress-tolerant hybrids. Nearly 80 local/regional seed companies and national seed organizations across 7 countries in Latin America have been partnering with CIMMYT for deploying climate-resilient improved maize varieties.

## Summary

Conventional maize breeding in the tropics has played an important role in increasing yields and food security (Renkow and Byerlee 2010; Edmeades et al. 2017; Zaidi et al. 2019). However, the rate of increase in productivity is currently not sufficient to meet future demands, particularly with increasing climate variability and population growth (Ray et al. 2015). This will be further compounded by the current COVID-19 pandemic; extreme poverty is projected to increase by 20% with the largest increase in SSA where 80 million more people may join the ranks of the extreme poor. In the face of these challenges, maize breeding programs in the tropics need to intensify efforts much more than ever before to increase genetic gains.

To increase genetic gains through maize breeding in the stress-prone tropics, and for enhancing the pace, precision and efficiency of breeding progress, judicious and effective integration of modern tools/strategies, especially high-density genotyping, high-throughput and precision phenotyping, DH technology, molecular marker-assisted and GS-based breeding, and knowledge-led decision-support systems, is vital. Besides climate resilience, maize varieties need to be enriched with nutritional quality to make a positive impact on the nutritional well-being of the consumers (Prasanna et al. 2020a, b, c). Accelerated replacement of climate-vulnerable maize varieties in the tropics (Cairns et al. 2018) will go a long way in improving the food and nutritional security and livelihoods of several million smallholders and their families.

## Abbreviations

AAA: Affordable, Accessible, Asian Drought-tolerant Maize
ASI: Anthesis-silking interval
CML: CIMMYT Maize Line
CRMA: Climate Resilient Maize for Asia
CGIAR: Consultative Group of International Agricultural Research
DH: Doubled haploid
DT: Drought tolerant
DTP: Drought-Tolerant Population
ESA: Eastern and Southern Africa
EBS: Enterprise Breeding System
FB: Forward breeding
GCA: General combining ability
GEBV: Genomic estimated breeding value
GWAS: Genome-wide association study
GS: Genomic selection
GEI: Genotype × Environment interaction
HTMA: Heat Tolerant Maize for Asia
IMTA: Improved Maize for Tropical Asia
IITA: International Institute of Tropical Agriculture
CIMMYT: International Maize and Wheat Improvement Center
IMIC-Asia: International Maize Improvement Consortium for Asia
MABC: Marker-assisted backcrossing
MARS: Marker-assisted recurrent selection
MT: Metric tons
MPS: Multiparent synthetic
NILs: Near-isogenic lines
OPVs: Open-pollinated varieties
PVP: Plant varietal protection
QTL: Quantitative trait loci
RCGS: Rapid-cycle genomic selection
RIL: Recombinant inbred line
SSA: Sub-Saharan Africa
mQTL: MetaQTL
SNP: Single nucleotide polymorphism
SCA: Specific combining ability
